# Special Issue: Back Pain in Children and Adolescents

**DOI:** 10.3390/children9050687

**Published:** 2022-05-10

**Authors:** Boris Zernikow, Michael Skovdal Rathleff

**Affiliations:** 1 German Paediatric Pain Centre, Children’s and Adolescents’ Hospital, 45711 Datteln, Germany; 2 Department of Children’s Pain Therapy and Paediatric Palliative Care, Faculty of Health, School of Medicine, Witten/Herdecke University, 58448 Witten, Germany; 3 Department of Health Science and Technology, Aalborg University, DK-9220 Aalborg, Denmark; misr@hst.aau.dk

***“What is the big deal about back pain in children and adolescents? Doesn’t it just go away if you give it time and rest?”***

The answer to this question is not straightforward, and there is a large knowledge gap surrounding the management of back pain in children and adolescents. One thing is certain though: back pain in children and adolescents greatly impacts the everyday lives of these young individuals and their medical care [1]. Like adults, the most common type of back pain in children and adolescents has no specific identifiable structure. However, there are also numerous “specific” congenital and acquired diseases that can cause back pain, and it is crucial not to miss treatable and potential harmful underlying conditions [2]. Knowledge of the differential diagnoses is important to the many professionals involved in the management of adolescent back pain. In many healthcare systems, the general practitioner may be the first point of contact, and then, depending on the clinical suspicion, there can be other disciplines that are secondary points of contact in the care system. These may include physiotherapists, orthopedists, oncologists, neurologists, radiologists, rheumatologists and others.

***“Why is there a need for special consideration of back pain in childhood and adolescence?”***

In the current absence of evidence, it is important to ensure that this population of young individuals are not exposed to under- (or over-) diagnosis or to over-treatment. This balance is truly difficult, as many questions regarding the origin and treatment of back pain remain unanswered. To guide clinical practice, Frosch et al. [3] have provided a consensus report based on evidence of the etiology, risk factors and diagnosis of back pain in children. Based on an extensive systematic literature search, grading of evidence and external review by experts from 14 different German medical associations, numerous evidence-based recommendations have been formulated regarding the diagnostic process of back pain in children [3]. The diagnosis of specific causes of disease is based on age-specific red flags. Especially in children under 10 years of age, age is already a risk factor for the presence of specific diseases in the occurrence of back pain, and so they require extended diagnostics.

***“What is the difference between back pain in younger children and in adolescents?”***

We see an increasing prevalence of “non-specific” back pain in adolescence [4,5]. Even at this age, non-specific back pain can take a chronic course and is therefore associated with a greater risk of impairment into adulthood [6]. Knowledge of the risk factors for non-specific back pain in adolescence is important, as they influence diagnosis and treatment [7]. In adult patients with non-specific back pain, common evidence-based treatments include reassurance, exercise therapy and medication; however, there is very limited evidence for medication use in childhood and adolescence [8,9]. In recent decades, the prevalence of non-specific back pain in children and adolescents has increased [10]. In order to counteract long-term impairments and the chronicity of the disease into adulthood, the prevention and age-appropriate treatment of back pain are particularly important [11].

Despite numerous scientific efforts to emphasize the problem of back pain in children and adolescents and to improve diagnostics and therapy, many research questions remain unanswered [12]. Future research should address the following topics in particular:

- Developing a reliable differential diagnosis of specific and non-specific back pain, including validating red flags;
- Clarifying the indications and procedure of imaging and multidisciplinary diagnostics;
- Optimizing non-drug treatments for non-specific back pain;
- Improving the prevention of back pain;
- Avoiding chronicity of back pain;
- Improving the self-management of non-specific back pain in children and adolescents.

In this Special Issue, we address these challenges of back pain in children and adolescents and give an overview of the current state of knowledge and future needs.
